# The Cytosolic Iron-Sulfur Cluster Assembly Protein MMS19 Regulates Transcriptional Gene Silencing, DNA Repair, and Flowering Time in *Arabidopsis*

**DOI:** 10.1371/journal.pone.0129137

**Published:** 2015-06-08

**Authors:** Yong-Feng Han, Huan-Wei Huang, Lin Li, Tao Cai, She Chen, Xin-Jian He

**Affiliations:** National Institute of Biological Sciences, Beijing, China; Universidade Federal de Vicosa, BRAZIL

## Abstract

MMS19 is an essential component of the cytoplasmic iron-sulfur (Fe-S) cluster assembly complex in fungi and mammals; the *mms19* null mutant alleles are lethal. Our study demonstrates that MMS19/MET18 in *Arabidopsis thaliana* interacts with the cytoplasmic Fe-S cluster assembly complex but is not an essential component of the complex. We find that MMS19 also interacts with the catalytic subunits of DNA polymerases, which have been demonstrated to be involved in transcriptional gene silencing (TGS), DNA repair, and flowering time regulation. Our results indicate that MMS19 has a similar biological function, suggesting a functional link between MMS19 and DNA polymerases. In the *mms19 *null mutant, the assembly of Fe-S clusters on the catalytic subunit of DNA polymerase α is reduced but not blocked, which is consistent with the viability of the mutant. Our study suggests that MMS19 assists the assembly of Fe-S clusters on DNA polymerases in the cytosol, thereby facilitating transcriptional gene silencing, DNA repair, and flowering time control.

## Introduction

Iron-sulfur (Fe-S) proteins are involved in fundamental cellular processes, such as electron transfer in mitochrondria and plastids, assembly of ribosomes, and DNA replication and repair [1–3]. The assembly of Fe-S clusters on their substrates depends on Fe-S cluster assembly proteins, most of which are evolutionarily conserved from microbes to mammals and plants. Fe-S cluster assembly is initiated in the mitochondria when a cysteine desulfurase complex Nfs1-Isd11 releases the sulfur required for iron-sulfur cluster formation [4]. The sulfur source is transferred to the mitochondrial ISC (iron-sulfur assembly complex) assembly machinery and may be exported to the cytosol by the ABC (ATP-binding cassette) transporter ATM1 for the assembly of cytosolic and nuclear Fe-S proteins [2]. In the cytosol of yeast, a diflavin reductase Tah18 first forms a complex with an Fe-S cluster-containing protein Dre2 for generation of stably inserted Fe-S clusters [5, 6], and subsequently Fe-S clusters are assembled on the scaffold complex CFD1-NBP35 [7]. Fe-S clusters are then released from NBP35 and transferred to target proteins, aided by the Fe-S protein NAR1 and the cytosolic iron-sulfur cluster assembly (CIA) complex [2, 7, 8]. NAR1 is likely to act as an adaptor between the CFD1-NBP35 complex and the CIA complex [7, 9]. A WD40-repeat protein CIA1 and a small acidic protein CIA2 are part of the CIA complex required for the maturation of Fe-S target proteins [10–12]. However, no CFD1 ortholog was found in *Arabidopsis*, where Fe-S clusters are assembled on the NBP35 homodimer [13].

MMS19 was primarily thought to be a component of the transcription factor TFIIH complex, which is involved in transcription, telomere length maintenance, and nucleotide excision repair in the nucleus [14–16]. Moreover, MMS19 was found in a chromatin silencing complex that contains MMS19, DOS2, RIK1, and CDC20 (the catalytic subunit of DNA polymerase ε) and is required for DNA replication, siRNA production, and heterochromatin assembly [17]. MMS19 is also required for proper chromosome segregation in human cells [18]. These findings suggest that MMS19 is involved in multiple processes in the nucleus. MMS19 was recently identified as a component of the CIA complex in yeast and mammals [3, 19, 20]. Using a yeast-two-hybrid assay, it was demonstrated that the *Arabidopsis* MMS19/MET18 (AT5G48120) physically interacts with CIA2/AE7 [8]. It is necessary to study how MMS19 regulates Fe-S cluster assembly and diverse nuclear processes in *Arabidopsis*.

DNA polymerases are central to the replication of double-stranded DNAs and are required for both heterochromatin silencing and DNA repair [21–23]. In *Arabidopsis*, several proteins involved in DNA replication and repair are required for transcriptional gene silencing (TGS) [23–25]. DNA polymerases α, δ, and ε are the main replicative DNA polymerases required for nuclear DNA replication in eukaryotes. DNA polymerase α is responsible for elongating the RNA primer with ~20 nucleotides. DNA polymerases δ and ε synthesize the lagging- and leading-strand DNAs, respectively [26]. The catalytic subunit of DNA polymerase ε contributes to the silencing of heterochromatin by associating with the silencing factors DOS2 and RIK1 in fission yeast [17]. In *Arabidopsis*, the null mutants of DNA polymerases α and ε are lethal [27–29], whereas their weak mutant alleles show pleotropic developmental phenotypes and are defective in DNA damage repair and TGS [30–32]. DNA polymerases α and ε are required for flowering repression, and their weak mutant alleles show an early flowering phenotype [29–31]. The DNA polymerases α, δ, and ε and the major DNA mutagenesis enzyme ζ were recently demonstrated to contain Fe-S clusters in the C-terminal domain (CTD) of their catalytic subunits in yeast [33]. Loss of Fe-S clusters in the DNA polymerase δ catalytic subunit Pol3 prevents the interaction of the catalytic subunit with the other subunits Pol31 and Pol32, suggesting that Fe-S clusters are necessary for the functioning of DNA polymerases. However, whether and how Fe-S clusters are assembled on DNA polymerases in plants remain unknown.

In this study, we demonstrate that MMS19 associates with the CIA complex in *Arabidopsis*, which is consistent with findings obtained with yeast and human cells [3, 19, 20]. Our gel filtration assay indicates, however, that MMS19 exists in a high-molecular-weight complex that differs from the other CIA complex components, suggesting a distinct role of MMS19 in *Arabidopsis*. Our study suggests that MMS19 in *Arabidopsis* enhances the assembly of Fe-S clusters on DNA polymerases and thereby contributes to TGS, DNA repair, and flowering time control.

## Results

### MMS19 associates with iron-sulfur assembly complex components and DNA polymerases

**Fig 1:**
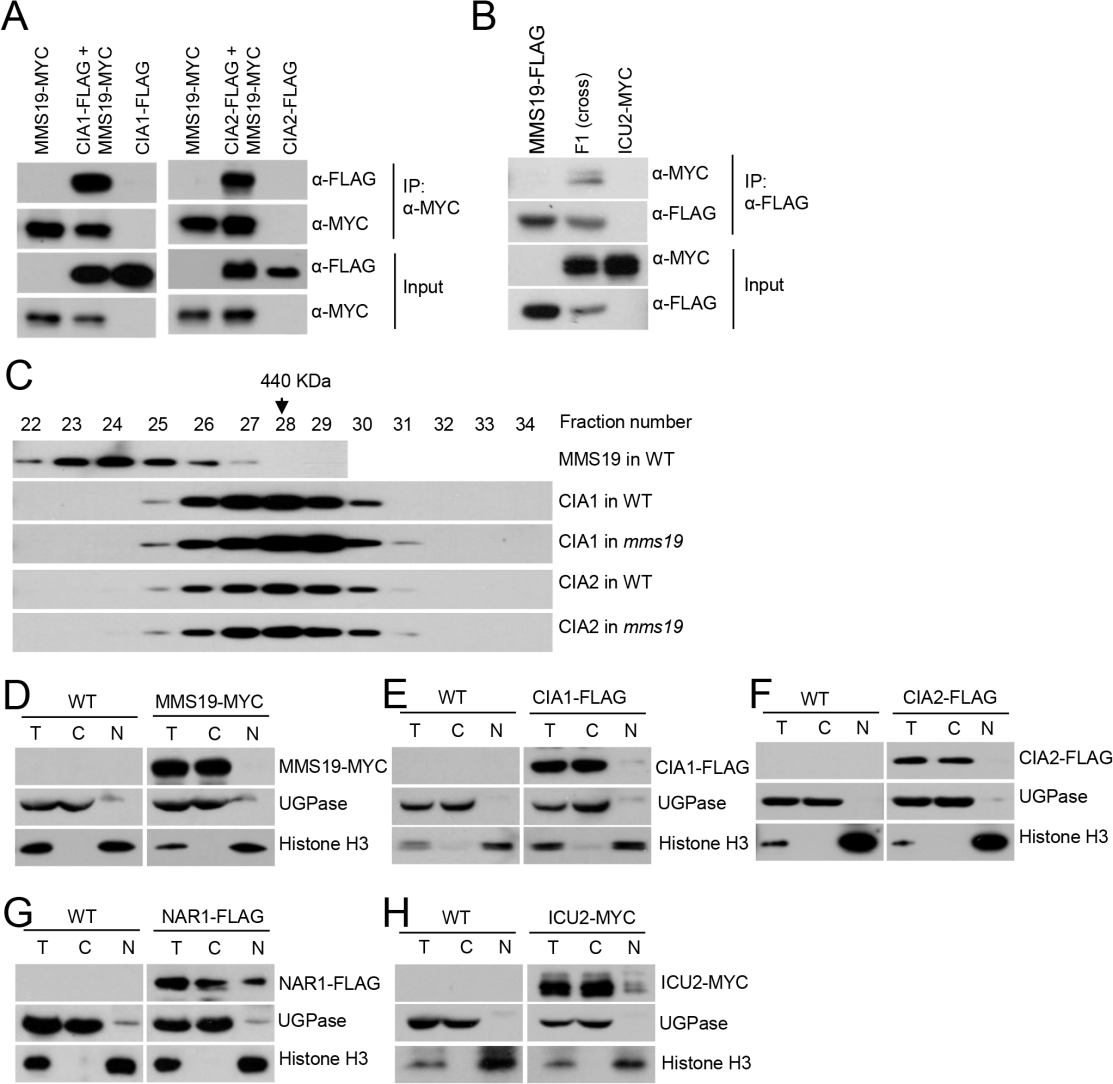
MMS19 interacts with the Fe-S assembly complex components CIA1 and CIA2 and the catalytic subunits of DNA polymerases in the cytoplasm. (A) MMS19 interacts with CIA1 and CIA2 *in vivo* as determined by co-IP. (B) MMS19 interacts with ICU2 *in vivo*. (C) Gel filtration analysis of MMS19, CIA1, and CIA2. The protein extracts were isolated from flowers of transgenic plants (*MMS19-Myc*, *CIA1-Flag*, and *CIA2-Flag*) in the wild-type or *mms19* mutant background and were loaded onto a Superose 6 10/300 GL column. The eluted fractions were run on an SDS-PAGE gel and subjected to Western blotting. The fraction numbers and sizes of standard proteins are shown. The subcellular localization of MMS19 (D), CIA1 (E), CIA2 (F), NAR1 (G), and ICU2 (H) as determined by nuclear-cytoplasmic fractionation. T: total extraction proteins, C: cytoplasmic proteins, N: nuclear proteins. MMS19, CIA1, and CIA2 mainly localized in the cytoplasm while NAR1 and ICU2 localized both in the cytoplasm and the nucleus. Histone H3 and UGPase were used as a nuclear marker and a cytoplasmic marker, respectively.

MMS19 was previously demonstrated to facilitate heterochromatin silencing in fission yeast, but the underlying mechanism is controversial [17, 19, 20]. Because MMS19 is conserved in eukaryotic organisms, it will be useful to determine whether and how MMS19 is involved in heterochromatin silencing in *Arabidopsis*. In *Arabidopsis*, MMS19 is a single protein and has no other homolog. We generated a construct harboring a native promoter-driven *MMS19*-*Myc* fusion sequence and transformed the construct into the *mms19* T-DNA mutant (*mms19-2*: Salk_147068) to obtain *MMS19-Myc* transgenic plants. We used anti-Myc antibody-conjugated beads to purify MMS19-associating proteins in the *MMS19-Myc* transgenic plants. Mass spectrometric analysis indicated that many proteins were identified by from both the wild-type control and the *MMS19-Myc* transgenic plants; these proteins are thought to be contaminants. The proteins shown in Table 1 were identified from the *MMS19-Myc* transgenic plants but not from the wild-type control. MMS19 is the most abundant protein in affinity purification of MMS19-Myc (Table 1). Moreover, other proteins were co-purified, and these included the cytosolic Fe-S assembly complex components CIA1, CIA2/AE7, and NAR1, as well as the catalytic subunits of DNA polymerases α, δ, and ε (ICU2, AT5G63960, and ABO4), suggesting that MMS19 may associate with these proteins (Table 1). We generated *CIA1-Flag* and *CIA2-Flag* constructs and transformed the constructs into *MMS19-Myc* transgenic plants, thereby obtaining plants harboring both *MMS19-Myc* and *CIA1-Flag* or *CIA2-Flag*. The interactions of MMS19 with CIA1 and CIA2 were confirmed by co-immunoprecipitation (co-IP) in these transgenic plants (Fig 1A). In addition, the interaction of MMS19 with ICU2 was confirmed by co-IP in the transgenic plants harboring both *MMS19-Flag* and *ICU2-Myc* transgenes (Fig 1B).

**Table 1:** Mass spectrometric analyses of MMS19, CIA1, and CIA2 affinity.

		MMS19 in WT		CIA1 in WT		CIA1 in *mms19*		CIA2 in WT		CIA2 in *mms19*	
AGI code	Protein	Mascot score	Unique peptides	Mascot score	Unique peptides	Mascot score	Unique peptides	Mascot score	Unique peptides	Mascot score	Unique peptides
AT5G48120	MMS19	5429	21	1658	21	0	0	3455	31	0	0
AT2G26060	CIA1	631	12	9870	65	16371	89	2157	16	2485	20
AT5G67100	ICU2	480	12	0	0	0	0	0	0	0	0
AT1G68310	CIA2	412	5	1542	10	2713	16	6373	15	6356	23
AT1G08260	ABO4	354	10	0	0	0	0	0	0	0	0
AT3G63460		347	7	140	2	0	0	182	3	69	1
AT5G63960	Pol δ	189	4	314	6	0	0	469	11	0	0
AT2G16950	TRN1	88	4	139	3	0	0	182	5	0	0
AT5G20020	RAN2	64	2	77	2	0	0	71	1	0	0
AT4G16440	NAR1	60	1	69	2	109	2	199	3	270	5
AT3G50845		0	0	416	7	294	5	0	0	0	0
AT3G11830		0	0	361	8	624	12	161	4	147	5
AT5G62600	MOS14	0	0	229	4	0	0	284	7	0	0

Total proteins were extracted from flowers and used for a gel filtration assay (Fig 1C). The result indicated that CIA1-Flag and CIA2-Flag were co-eluted at ~440 KD (Fig 1C). Previous studies demonstrated that the homologs of CIA1 and CIA2 in fungi and animals exist in a conserved CIA complex [10–12]. Our gel filtration results demonstrated that CIA1 and CIA2 are present in complexes of the same size (Fig 1C), suggesting that the *Arabidopsis* CIA1 and CIA2 also exist in a conserved CIA complex. We introduced the *CIA1-Flag* and *CIA2-Flag* constructs into the *mms19* mutant background and found that the size of CIA1- and CIA2-containing complex is not affected in the *mms19* mutant as compared to that in the wild type (Fig 1C), suggesting that MMS19 is not an essential component of the CIA complex even though it associates with CIA1 and CIA2. As determined by the gel filtration assay, we found that although the elution of the MMS19-containing complex is partially overlapped with the CIA1- and CIA2-containing complex, the MMS19-containing complex is mostly eluted in high-molecular-weight fractions in which CIA1 and CIA2 are not present (Fig 1C). While MMS19 associates with the CIA complex components CIA1 and CIA2, it may form a high-molecular-weight complex that is different from the CIA complex. As determined by affinity purification of MMS19 (Table 1), the high-molecular-weight MMS19-containing complex may contain DNA polymerases and other uncharacterized proteins. Whether and how these proteins act in Fe-S cluster assembly remain to be studied in future.

DNA polymerases α, δ, and ε are the main replicative DNA polymerases in eukaryotes. The catalytic subunits of the three polymerases in fission yeast have been demonstrated to be Fe-S proteins [33]. In our results, affinity purification of MMS19 co-purified all three polymerase catalytic subunits including ICU2, ABO4/TIL1, and AT5G63960, whereas affinity purification of CIA1 and CIA2 co-purified only AT5G63960 (Table 1). In the *mms19* mutant background, however, AT5G63960 was undetectable in the CIA1 and CIA2 affinity-purified proteins (Table 1). MMS19 may facilitate the interaction of CIA1 and CIA2 with the catalytic subunit of DNA polymerase δ.

### Subcellular localization of MMS19, CIA1, CIA2, and NAR1

Although MMS19 was recently found to be associated with Fe-S assembly in the cytoplasm [8, 19, 20], MMS19 was previously reported to directly associate with chromatin and act in heterochromatin silencing in the nucleus [17]. Subcellular localization of MMS19 was determined in *MMS19-Flag* transgenic plants. Nuclear proteins and cytosolic proteins were extracted from the transgenic plants and subjected to Western blotting assay. The antibodies against UGPase and histone H3 were used as markers for cytoplasmic and nuclear proteins, respectively. The results indicated that MMS19 is localized in the cytoplasm but not in the nucleus (Fig 1D). Subcellular localization of CIA1, CIA2, and NAR1 was determined in *CIA1-Flag*, *CIA2-Flag*, and *NAR1-Flag* transgenic plants, respectively. We found that CIA1 and CIA2 are cytoplasmic proteins and that NAR1 exists in both the cytoplasm and the nucleus (Fig 1E, 1F and 1G). The localization of MMS19, CIA1, and CIA2 in the cytoplasm is consistent with the inference that the CIA complex is responsible for Fe-S cluster assembly in the cytoplasm [8, 19, 20]. The yeast NAR1 is predominantly localized in the cytoplasm [9], whereas the *Arabidopsis* NAR1 is present in both the cytoplasm and the nucleus (Fig 1G). NAR1 was thought to transfer Fe-S clusters from the Fe-S cluster scaffold protein NBP35 to the CIA complex at an early step of the cytosolic Fe-S cluster assembly in yeast [10]. In *Arabidopsis*, the partially nuclear localization of NAR1 is probably required for Fe-S cluster assembly on nuclear Fe-S proteins.

Among the three DNA polymerases identified by affinity purification of MMS19 (Table 1), ICU2 and ABO4 were previously characterized [32]. However, it is difficult to understand how the cytoplasmic MMS19 interacts with ICU2 and ABO4, which are primarily thought to be located in the nucleus (Table 1 and Fig 1B). To determine the subcellular localization of ICU2 and ABO4, we primarily tried to generate tagged transgenic plants for both *ICU2* and *ABO4*. Because the size of *ABO4* is long (~16 Kb), we failed to clone *ABO4*. Thus, we obtained only *ICU2-Myc* transgenic plants. The *ICU2-Myc* signal is predominantly present in the cytoplasm rather than in the nucleus (Fig 1H), suggesting that the interaction of ICU2 with MMS19 occurs in the cytoplasm.

### MMS19 is involved in transcriptional gene silencing

**Fig 2:**
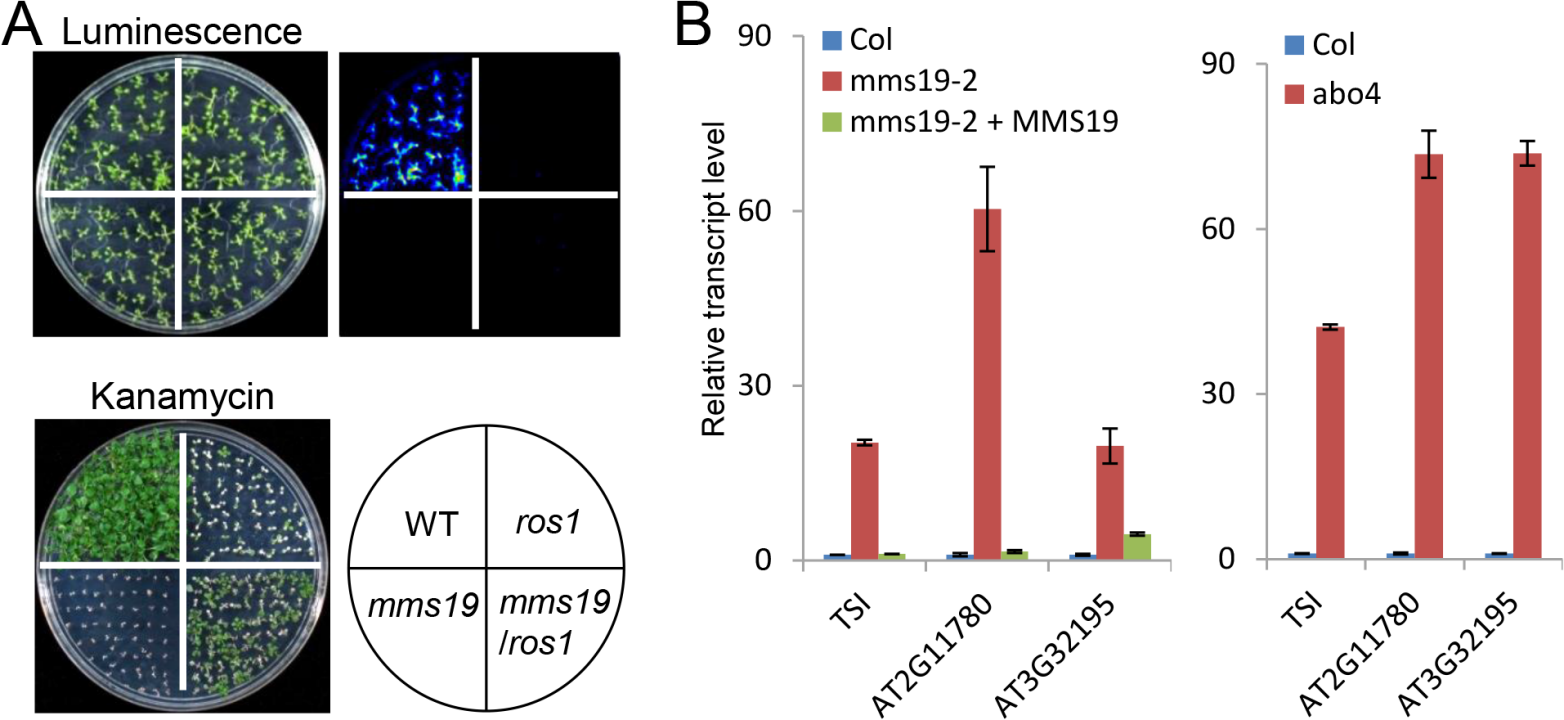
Silencing of *35S-NPTII* transgene and endogenous transposable elements and other loci is affected in the *mms19* mutant. (A) The effect of *mms19* on the silencing of *RD29A-LUC* and *35S-NPTII* transgenes. Each genotype harboring the *RD29A-LUC* and *35S-NPTII* transgenes was grown on MS medium for 14 days followed by cold treatment for 2 days at 4 °C. The treated seedlings were sprayed with luciferin for luminescence imaging. The seedlings were grown on MS medium supplemented with 150 mg/L kanamycin for 20 days and photographed. (B) The *mms19* and *abo4* mutants release the silencing of transposable elements. The transcript levels of the transposable element genes *TSI*, *AT2G11780*, and *AT3G32195* were detected in the wild type, *mms19-2* and its complementation line, and *abo4* by quantitative RT-PCR. *ACT7* was used as an internal control for normalization. Quantitative RT-PCR experiments were biologically repeated three times with similar results. Showing is the result of three technical replicates from one representative experiment.

The catalytic subunits of the replicative DNA polymerases α, δ, and ε were co-purified by affinity purification of MMS19 (Table 1 and Fig 1B). Thus, we predicted that MMS19 is functionally associated with the DNA polymerases. Two individual *mms19* mutants, *mms19-1* (salk_121963) and *mms19-2* (salk_147068) were obtained from *Arabidopsis* Stock Center. The transcript level of *MMS19* was not detected in the two mutants by quantitative RT-PCR, indicating that the two *mms19* mutants are null allele mutants (S1 Fig). In *Arabidopsis*, the catalytic subunits of the DNA polymerases α and ε (ICU2 and ABO4) were identified as regulators of TGS [30, 32]. The *RD29A* promoter-driven luciferase transgene (*RD29A-LUC*) and the accompanied *35S* promoter-driven *NPTII* transgene (*35S-NPTII*) are expressed well in the C24 wild-type background but are silenced when the DNA demethylase gene *ROS1* is depleted in the *ros1* mutant [34]. In the *ros1icu2* and *ros1abo4* double mutants, the expression of *35S-NPTII* but not of *RD29A-LUC* is reactivated, suggesting that ICU2 and ABO4 are required for the silencing of *35S-NPTII* in the *ros1* mutant background [30, 32]. To determine whether MMS19 is required for the silencing of *RD29A-LUC* and *35S-NPTII* in the *ros1* mutant background, we generated the *ros1mms19* double mutant harboring both *RD29A-LUC* and *35S-NPTII* by crossing. We found that although *35S-PTII* exists in both the *ros1* single mutant and the *ros1mms19* double mutant, the *ros1mms19* double mutant is much more resistant to kanamycin than the *ros1* single mutant, suggesting that *mms19* releases the silencing of *35S-NPTII*. Meanwhile, the *mms19* mutation has no effect on the silencing of *RD29A-LUC* as determined by luminescence imaging (Fig 2A). The results suggest that MMS19 is required for the DNA polymerase-mediated transgene silencing.

*TSI* (*transcriptional silencing information*) loci are the pericentromeric *Athila* retrotransposons that are subjected to transcriptional silencing in the wild-type *Arabidopsis* [35]. The silencing of *TSI* is significantly released in the *icu2* and *abo4* mutants [30, 32]. We found that the silencing of *TSI* is also released in the two individual *mms19* mutants and that this effect is rescued by the native promoter-driven *MMS19-Myc* transgene (Fig 2B and S2 Fig). Moreover, we found that the silencing of the transposon genes *AT2G11780* and *AT3G32195* is released by *mms19* as well as by *abo4-1* (Fig 2B and S2 Fig). These results demonstrate that MMS19 is functionally associated with the DNA polymerases α and ε in TGS.

### MMS19 and ABO4 have a similar effect on the expression of genes and transposable elements (TEs)

**Fig 3:**
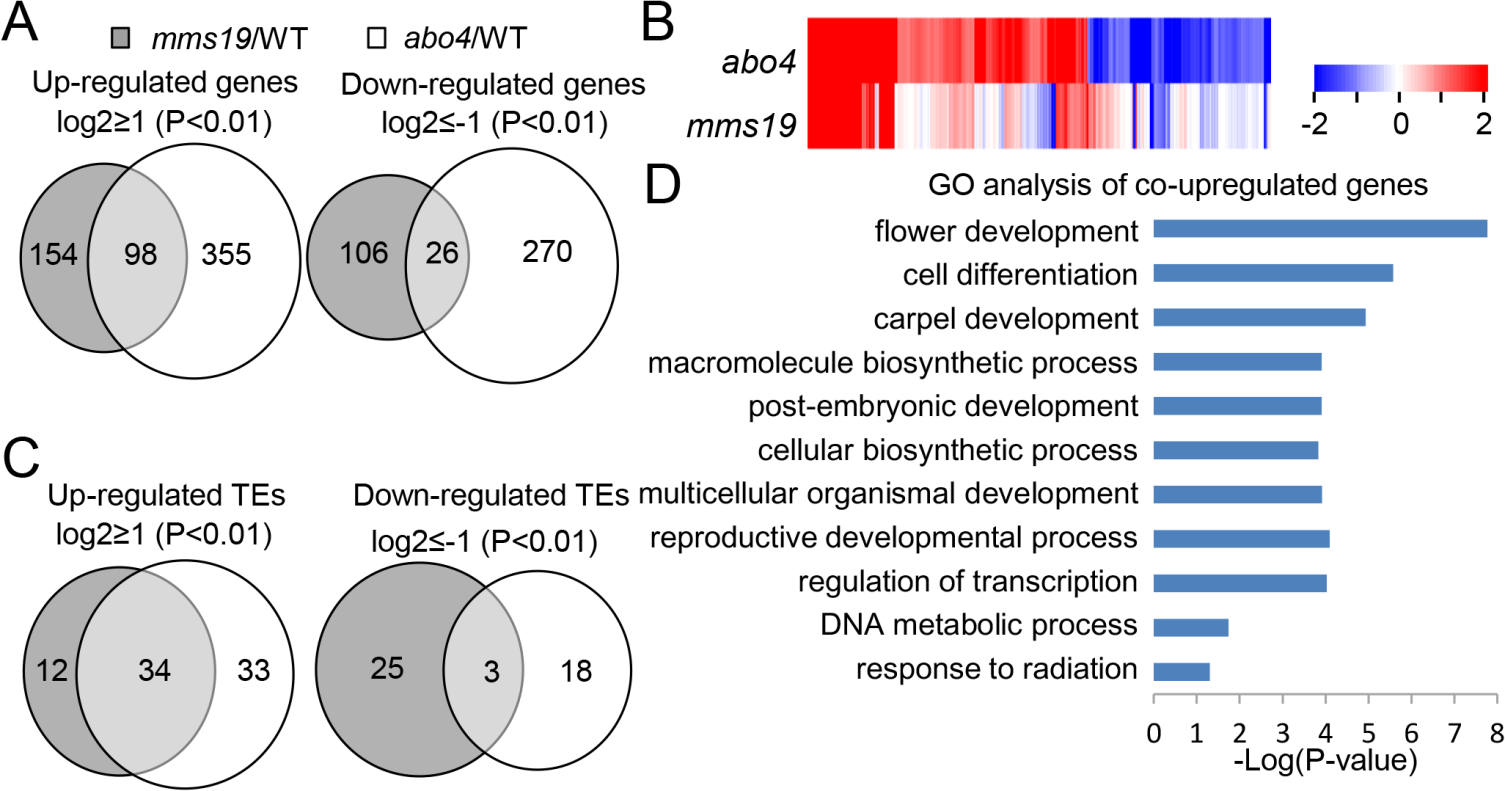
The effect of *mms19* on the transcriptome as determined by RNA-seq analyses. (A) Differentially expressed genes in *mms19* and *abo4* mutants relative to the wild type are shown by Venn diagrams. (B) Differentially expressed genes in *mms19* and *abo4* mutants relative to the wild type are shown by heat maps. (C) Differentially expressed TEs in *mms19* and *abo4* mutants relative to the wild type are shown by Venn diagrams. (D) Gene Ontology (GO) analysis of co-upregulated genes in *mms19* and *abo4* mutants. The lengths of bars represent statistical values of gene enrichment in the indicated biological processes. The biological processes are listed only when their genes are significantly (P<0.05) enriched.

Because of the physical and functional association between MMS19 and ABO4, we performed RNA-sequencing (RNA-seq) analyses to determine whether MMS19 and ABO4 have a similar effect on the expression of genes and TEs. Although null mutant alleles of *ABO4* are embryonically lethal [27, 28], the weak missense mutant allele *abo4-1* is viable and is defective in TGS [30]. Thus, we used RNA-seq analyses to compare the effect of the null *mms19-2* mutation and the weak *abo4-1* mutation on the expression of genes and TEs. In total, 5.86x10^7^, 5.37x10^7^, and 5.12x10^7^ reads were obtained from the RNA libraries of the wild type, *mms19-1*, and *abo4-1*, respectively, and most of the reads are uniquely matched on the Tair10 *Arabidopsis* genome. The RNA sequencing data indicated that 252 and 453 genes are significantly upregulated (log_2_(fold-change)>1; P<0.01) in *mms19* and *abo4*, respectively (Fig 3A, S1 and S2 Tables). Of the 252 genes upregulated in *mms19*, 98 (98/252 = 38.9%) are also upregulated in *abo4* (Fig 3A). The RNA sequencing data also indicated that 132 and 296 genes are significantly downregulated (log_2_(fold-change)<-1; P<0.01) in *mms19* and *abo4*, respectively (Fig 3A, S1 and S2 Tables). Of the 132 genes downregulated in *mms19*, 26 (26/132 = 19.7%) are also downregulated in *abo4* (Fig 3A, S1 and S2 Tables). The number of target genes shared by MMS19 and ABO4 is significantly greater than expected by random (P<0.01), suggesting that the two components have a similar effect on gene expression. Heat maps further confirmed that differentially expressed genes caused by *mms19* and *abo4* are positively correlated (Fig 3B), supporting a functional connection between MMS19 and ABO4. We randomly selected seven co-upregulated genes and three co-downregulated genes for validation by quantitative RT-PCR. The results demonstrated that the RNA-seq data are reliable (S3 Fig).

We compared the effect of *mms19* and *abo4* on the silencing of TEs based on the RNA-seq data. The results indicated that 46 and 67 TEs are upregulated in *mms19* and *abo4*, respectively (Fig 3C, S3 and S4 Tables). Among the 46 TEs upregulated in *mms19*, most (34/46 = 73.9%) are also upregulated in *abo4* (Fig 3C). We randomly selected three co-upregulated TEs and validated their expression by quantitative RT-PCR; the results confirmed that all three TEs are co-upregulated (S3 Fig). These results suggest that MMS19 and ABO4 have a similar effect on the silencing of TEs. The numbers of downregulated TEs in *mms19* and *abo4* are 28 and 21, respectively (Fig 3C, S3 and S4 Tables). The downregulation of genes and TEs in *mms19* and *abo4* is probably due to an indirect effect of *mms19* and *abo4* on the expression of some uncharacterized transcriptional regulators.

Previous studies reported that *icu2* and *abo4* are defective in DNA repair and flowering time control [29, 30, 32]. Our RNA-seq data indicated that several DNA repair-related genes and flower development-related genes are upregulated not only in *mms19* and but also in *abo4* (S1 and S2 Tables). To characterize the common target genes shared by MMS19 and ABO4, we classified the co-upregulated genes by Gene Ontology (GO). The results suggest that flower development-related genes and DNA metabolic process genes are enriched (Fig 3D), which is consistent with the function of MMS19 and ABO4 in flowering time control and DNA metabolism. Additionally, we found that the common target genes of MMS19 and ABO4 are also highly correlated with several other biological processes (Fig 3D). These results will be valuable for understanding additional biological functions of MMS19 and DNA polymerase ε in *Arabidopsis*.

### MMS19 is required for DNA repair

**Fig 4:**
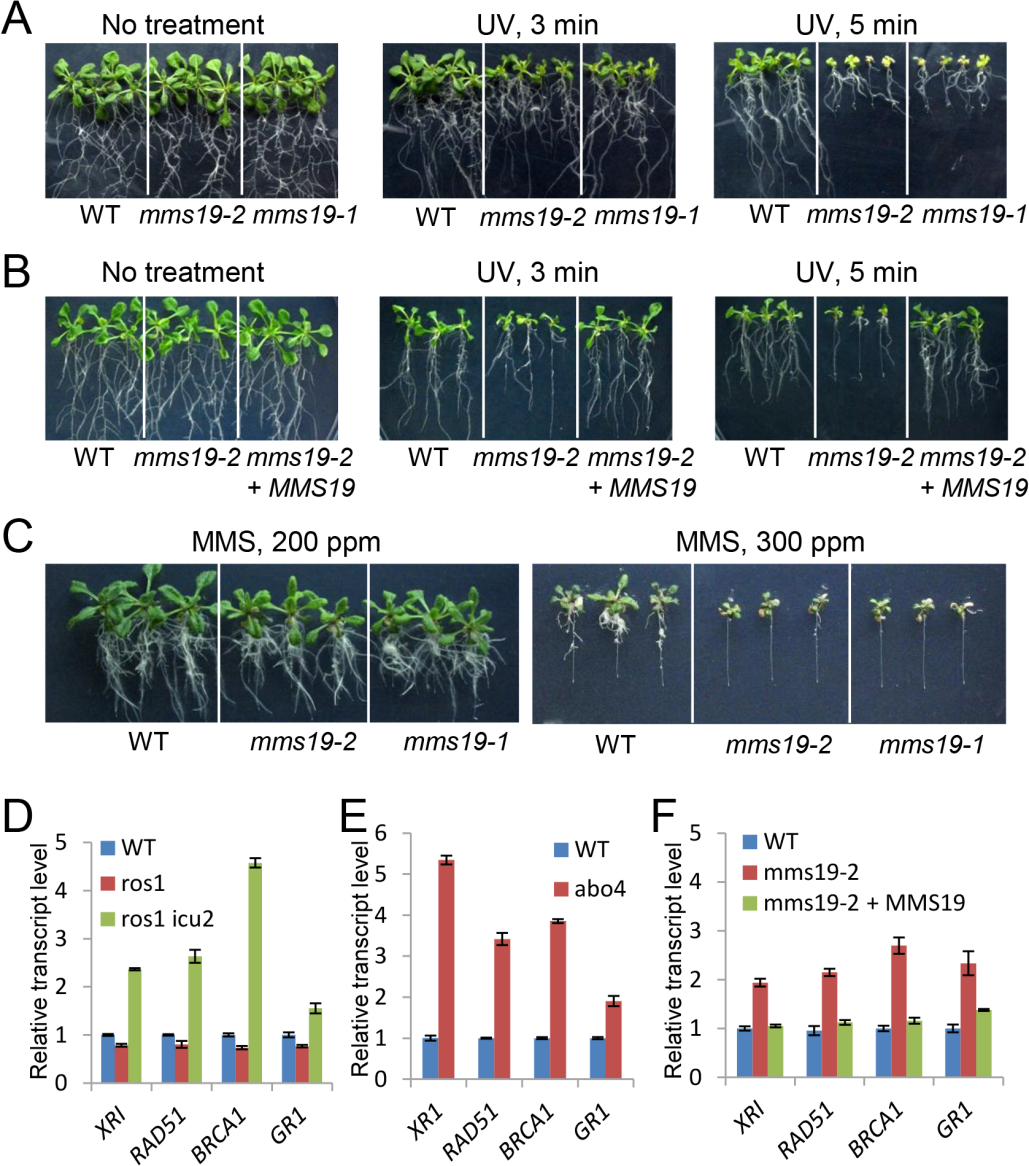
The *mms19* mutants are sensitive to DNA damage and constitutively express a high level of DNA repair-related genes. (A) The *mms19* mutants are more sensitive to UV-B treatment than the wild type. Five-day-old seedlings of the wild type and *mms19* mutants were treated with UV-B light (1 kJ/m^2^) for different times and then grown with 16-h-light and 8-h-dark at 22° for 5–7 days before being photographed. (B) The sensitivity of the *mms19-2* mutant to UV-B treatment was rescued by the *MMS19-Myc* transgene in T2 transgenic plants. (C) The *mms19* mutants are more sensitive than the wild type to the DNA-damaging reagent methyl methanesulfonate (MMS). Quantitative RT-PCR results indicate that DNA repair-related genes were up-regulated in *ros1icu2* (D), *abo4* (E), and *mms19* (F) mutants compared to their respective control plants. The seedlings were grown on MS medium plates for ~10 days under long-day-condition and then harvested for RNA isolation. Quantitative RT-PCR experiments were biologically repeated with similar results, and three technical replicates of one representative experiment were shown. Error bars represent the standard deviation (SD).

The two catalytic subunits of DNA polymerases, ICU2 and ABO4, are required for DNA repair [23, 30, 32]. That DNA repair-related genes present similar regulation in *mms19* and *abo4* mutants as determined by our RNA-seq data (S1 and S2 Tables, Fig 3D) suggested that MMS19 may also be required for DNA repair. Five-day-old seedlings of the wild-type control and the two independent *mms19* mutants (*mms19-1* and *mms19-2*) were subjected to UV treatment and then grown on vertical MS medium plates for 10 days. Growth was more severely retarded for *mms19-1* and *mms19-2* than for the wild type (Fig 4A), suggesting that the *mms19* mutants are sensitive to UV treatment. Furthermore, the full-length *MMS19* transgene restored the UV resistance of the *mms19-2* mutant (Fig 4B). To confirm the function of MMS19 in DNA repair, we determined the sensitivity of *mms19-1* and *mms19-2* to the DNA-damaging regent MMS (methyl methanesulfonate). The result demonstrated that the two *mms19* mutants are sensitive to MMS, especially when the concentration of MMS is increased to 300 ppm (Fig 4C). Therefore, like DNA polymerases α and ε, MMS19 is required for DNA repair.

Previous studies reported that several DNA repair-related genes are upregulated by the mutations of the DNA polymerase genes *ICU2* and *ABO4* [30, 32]. *ROS1* is responsible for DNA demethylation and repression of transgene silencing. In the *ros1* mutant background, *35S-NPTII* transgene is silenced. The mutation of *ICU2* in the *ros1* mutant background releases the silencing of *35S-NPTII* transgene. The function of ICU2 in regulation of DNA repair-related genes was detected not only in the *ros1* mutant background but also in the wild-type background [30]. Thus, the *ros1icu2* mutant was used to determine the effect of *icu2* on the expression of DNA repair-related genes (Fig 4D). Consistent with the previous studies, our quantitative RT-PCR results indicated that the transcript levels of the DNA repair-related genes *XRI*, *RAD51*, *BRCA1*, and *GR1* are upregulated by *icu2* and *abo4* (Fig 4D and 4E). The effect of *mms19* on the expression of the DNA repair-related genes was determined by quantitative RT-PCR. The results indicated that the DNA repair-related genes including *XRI*, *RAD51*, *BRCA1*, and *GR1* are induced in the *mms19-1* and *mms19-2* mutants (Fig 4F and S2 Fig). Moreover, the full-length *MMS19-Myc* transgene restores the transcript levels of these genes in the *mms19-2* mutants (Fig 4F). We further determined the effect of the *mms19* mutations on the expression of these genes under genotoxic conditions (S4 Fig). The results indicated that *XRI*, *RAD51*, *BRCA1*, and *GR1* are induced by MMS treatment not only in the wild type but also in the *mms19* mutants (S4 Fig), which is consistent with the notion that these genes are induced by DNA double-strand breaks caused by MMS. Whether or not the seedlings were treated with MMS, the *mms19* mutations affect the expression of DNA repair-related genes (S4 Fig). These results suggest that MMS19 is functionally associated with the DNA polymerases α and ε in DNA repair.

### MMS19 is involved in flowering time control

**Fig 5:**
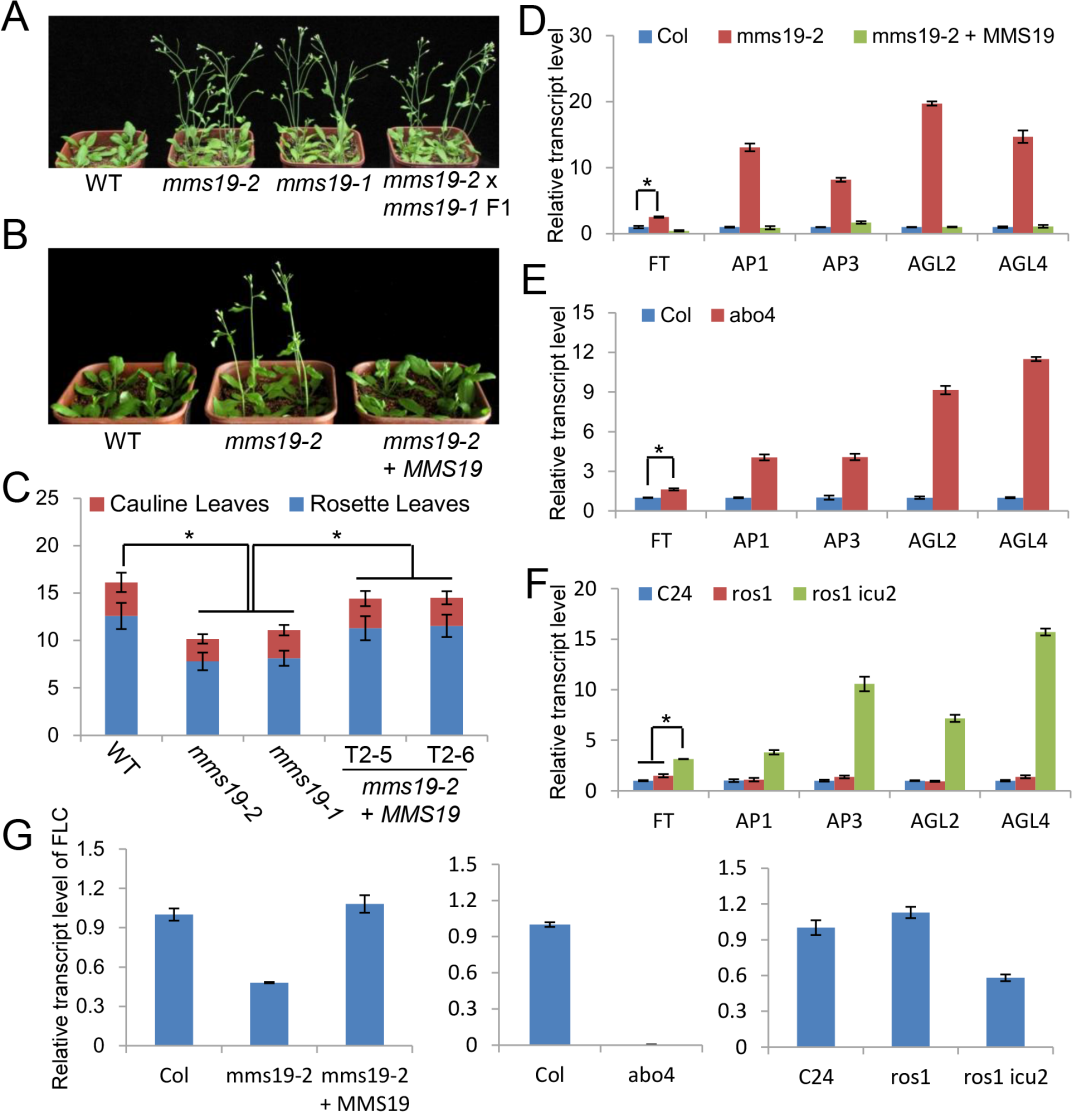
The *mms19* mutants cause an early flowering phenotype by affecting the expression of flowering-related genes. (A) The early flowering phenotype of *mms19* mutants in standard long-day conditions (16-h-light and 8-h-dark at 22°C). (B) The early-flowering phenotype was restored by the *MMS19-Myc* construction in *mms19-2*. (C) The statistics of total leaf numbers upon flowering under long-day conditions in the wild type, the *mms19* mutants and the complementation lines. T2-5 and T2-6 were two randomly selected individual *MMS19-MYC* transgenic lines in T2 generation. At least 30 individual plants were counted. Error bars stand for SD. Asterisks indicate significant difference as determined by the *t*-test (P<0.05). Numbers of rosette and cauline leaves are indicated by blue and red bars, respectively. (D) Leaf numbers under long-day conditions with or without vernalization. (E) Leaf numbers under short-day conditions. (F), (G) and (H) The effect of *mms19*, *abo4*, and *icu2* on the expression of flowering-related genes as determined by quantitative RT-PCR. *ACT7* was amplified as an internal control. The quantitative RT-PCR experiments were biologically repeated for three times and indicated similar results. A representative repetition is shown. Error bars represent SD. Asterisks show significant difference as determined by the *t*-test (P<0.05).

An early flowering phenotype was previously reported in *icu2* and *abo4* [30, 32]. We found a similar early flowering phenotype in two individual *mms19* mutants, *mms19-1* and *mms19-2* (Fig 5A). An allelic test indicated that the F1 plants obtained from the crossing of *mms19-1* and *mms19-2* flower as early as their parent plants (Fig 5A), confirming that the early flowering phenotype is caused by the *mms19* mutations in *mms19-1* and *mms19-2*. Under long-day conditions (16 h light/8 h dark), the wild-type plants have an average of 12.6 rosette leaves upon flowering, while the two individual *mms19* mutants flower with ~8.0 rosette leaves (Fig 5B and 5C). The full-length *MMS19-Myc* construct was transformed into the *mms19-2* mutant for complementation testing. The *MMS19-Myc* transgene in two individual *MMS19-Myc* transgenic lines almost restores the flowering time of *mms19* to the wild-type level (Fig 5B and 5C). These results suggest that flowering time is regulated not only by *icu2* and *abo4* but also by *mms19*.

Flowering time is regulated by various environmental and endogenous signaling pathways, which include vernalization, photoperiod, and autonomous pathways [36, 37]. When a vernalization treatment (4°C for 30 days) was applied, flowering was affected in the *mms19* mutants as well as in the wild type (Fig 5D), indicating that the role of MMS19 in flowering time control is independent of the vernalization pathway. When photoperiod was changed from long-day (16-h-light and 8-h-dark) to short-day (8-h-light and 16-h-dark) conditions, the flowering time was delayed in both the wild type and the *mms19* mutants (Fig 5E). However, the effect of the photoperiod change on flowering time is much smaller in the *mms19* mutants than in the wild type (Fig 5E). The result suggests that the involvement of MMS19 in flowering time is regulated by photoperiod.

FLC acts as a central floral repressor through which the flowering time is regulated by vernalization, photoperiod, and autonomous pathways [37, 38]. FT is a key floral integrator and activates a number of downstream genes required for flower development [36, 39]. The expression of *FT* is directly repressed by *FLC* [40, 41]. The early flowering phenotype of *icu2* and *abo4* is related to the expression of *FT* [29, 30]. We determined whether the early flowering phenotype of the *mms19* mutant is related to the expression of *FLC*, *FT*, and other floral integrator genes. Quantitative RT-PCR indicated that the floral integrator genes *FT*, *AP1*, *AP2*, *AGL2*, and *AGL4* are markedly upregulated in the *mms19* mutant and that the introduced *MMS19-Myc* transgene restores the expression of these genes to wild-type levels (Fig 5F). Moreover, the expression of these floral integrator genes is upregulated by both *icu2* and *abo4* as determined by quantitative RT-PCR (Fig 5G and 5H). Interestingly, the expression of the floral repressor gene *FLC* is not only downregulated by *mms19* but also by *icu2* and *abo4* (Fig 5F, 5G and 5H). These results suggest that the involvement of MMS19 in flowering time control is likely related to the DNA polymerases α and ε.

### MMS19 is involved in Fe-S assembly on DNA polymerase α

**Fig 6:**
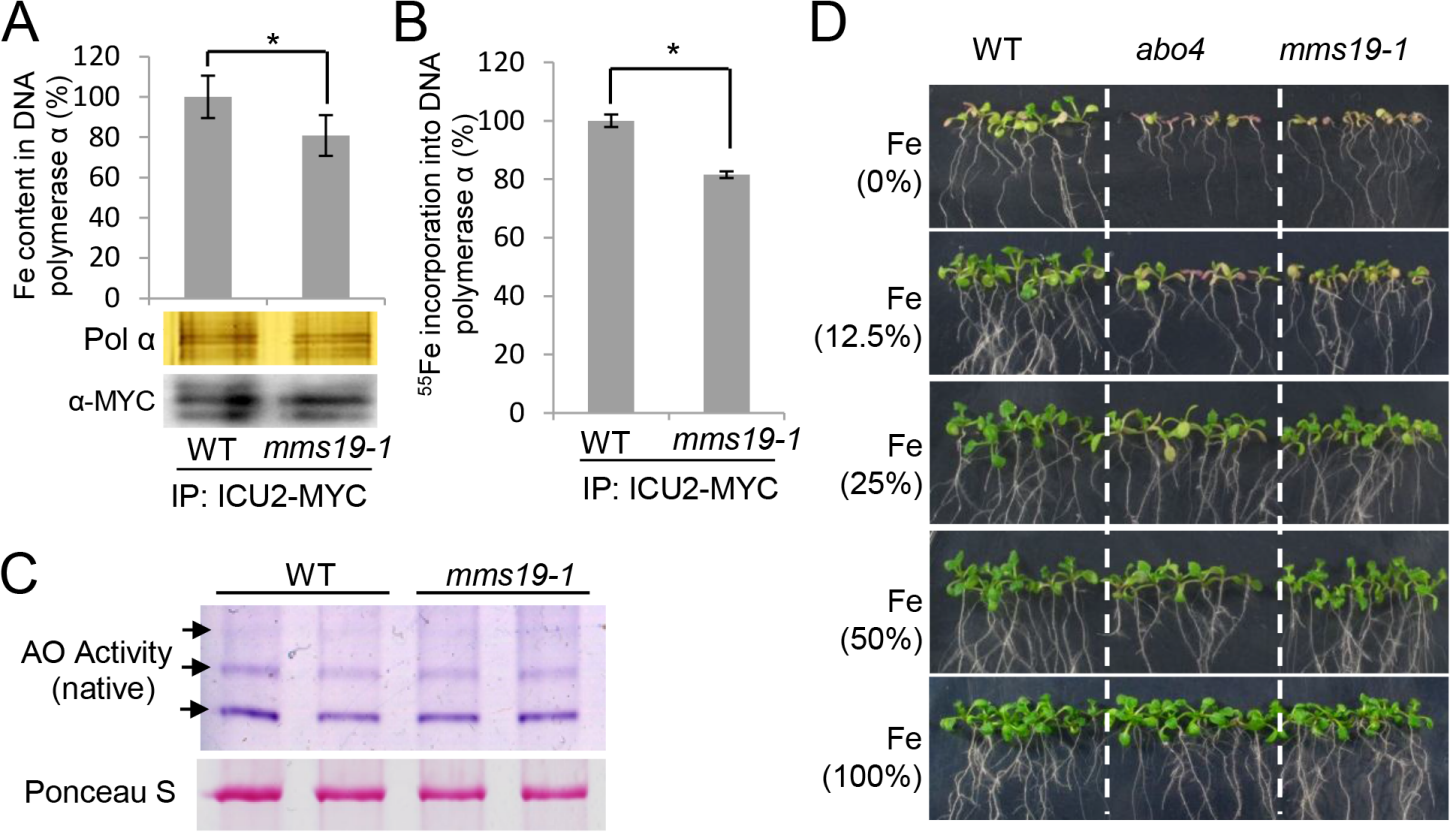
The *mms19* mutant incorporates a reduced level of Fe in ICU2 and is sensitive to Fe absence. (A) Fe content of ICU2 in the wild type and *mms19*. The protein extraction was affinity purified by anti-Myc antibody-coupled beads and eluted three times by 0.1 M ammonium hydroxide. The three elutions were combined and subjected to Fe content detection by ICP-MS (Themo ICP-MS XII). The affinity purified ICU2-Myc in the wild type and the *mms19* mutant was separated by SDS-PAGE and subjected to silver staining and western blotting as controls. The results of three biological repeats are indicated. Error bars show the SD. Asterisks indicate significant differences as determined by the *t*-test (P<0.05). (B) ^55^Fe incorporation into ICU2 in the wild type and the *mms19* mutant. The same affinity purification method was used as in (A), and the radioactivity of ^55^Fe in ICU2 was measured by liquid scintillation counting. Triplicates were performed. Error bars show the SD. Asterisks indicate significant differences as determined by the *t-*test. (C) The activities of aldehyde oxidase (AO) isozymes in wild-type and *mms19* mutant seedlings. The purple bands indicated by arrows represent the activities of AO proteins. The staining by Ponceau S is shown as an equal protein loading control. (D) The *mms19* and *abo4* seedlings were more sensitive than wild-type seedlings to reduced FeSO_4_ concentrations. Seedlings were photographed after they had grown for 10 days on MS medium plates with different FeSO_4_ concentrations. 100%, 50%, 25%, and 12.5% represent 2.78, 1.39, 0.70, and 0.35 g/l of FeSO4.7H2O in MS medium.

The MMS19 orthologs in yeast and mammals are thought to act as canonical components of the cytosolic Fe-S assembly complexes [19, 20]. Because the catalytic subunits of DNA polymerases α, δ, and ε in yeast contain Fe-S clusters [33], the finding of the interaction between MMS19 and DNA polymerases in *Arabidopsis* motivated us to determine whether MMS19 is involved in the assembly of Fe-S clusters on the catalytic subunits of DNA polymerases. We transformed an *ICU2-Myc* construct into the wild type and obtained a transgenic line harboring a single copy of the *ICU2-Myc* transgene. This *ICU2-Myc* transgenic line was crossed to the *mms19* mutant to introduce the *ICU2-Myc* transgene into the *mms19* mutant background. We performed quantitative PCR to confirm that the same copy of *ICU2-Myc* transgene is present in the wild type and the *mms19* mutant (data not shown). The amount of ICU2-Myc protein was determined by both silver staining and western blotting (Fig 6A). The results suggest that the ICU2-Myc protein level is comparable between the wild type and the *mms19* mutant (Fig 6A). By ICP mass spectrometric assay, we found that the Fe content in DNA polymerase α was less in the *mms19* mutant than in the wild type (Fig 6A), suggesting that the Fe-S assembly of DNA polymerase α is affected in the *mms19* mutant. To confirm the effect of *mms19* on the Fe-S assembly of DNA polymerase α, we incubated *Arabidopsis* seedlings in radioactive ^55^Fe-labelled liquid MS medium and determined whether the incorporation of ^55^Fe in the DNA polymerase α is affected in the *mms19* mutant. Liquid scintillation counting indicated that ^55^Fe incorporation is lower in the *mms19* mutant than in the wild type (Fig 6B). The two different experiments demonstrated that Fe incorporation into ICU2 is reduced but not blocked in the *mms19* mutant, suggesting that MMS19 is not an essential component of the Fe-S assembly complex but acts as an enhancer of the Fe-S assembly of DNA polymerase α. We cannot completely exclude the possibility, however, that the ICU2-Myc protein level is slightly reduced in the *mms19* mutant compared to that in the wild type. If it is true, the lower amount of Fe content in the *mms19* mutant is at least partially caused by the effect of *mms19* on the accumulation of the ICU2-Myc protein.

We simultaneously tested the effect of *mms19* on the activities of aldehyde oxidases (AOs), which carry 2[2Fe-2S] clusters [42]. The enzymes catalyze the conversion of sirtinol into an auxin analog, and the Fe-S cluster assembly is required for their enzymatic activity [42]. In-gel staining indicated that the enzymatic activities of all three AO isoforms are unchanged in the *mms19* mutant relative to the wild type (Fig 6C), suggesting that MMS19 is not required for the Fe-S cluster assembly of the three AO isoforms. It is possible that MMS19 is involved in Fe-S cluster assembly on a subset of Fe-S target proteins.

To confirm the involvement of Fe in the function of MMS19 and DNA polymerases, we incubated wild-type, *mms19*, and *abo4* seedlings on MS medium plates containing different concentrations of FeSO_4_ (Fig 6D). The results indicated that the growth of *mms19* and *abo4* is retarded compared to that of the wild type when FeSO_4_ is reduced in the MS medium, whereas both *mms19* and *abo4* grow as well as the wild type on the control MS medium (Fig 6D). The sensitivity of *mms19* and *abo4* to Fe demonstrated that Fe is related to the function of MMS19 and ABO4 in *Arabidopsis*. According to a recent research with yeast, the catalytic subunits of DNA polymerases are Fe-S proteins, and their catalytic functions require Fe-S clusters [33]. Because DNA polymerases are conserved in eukaryotes, MMS19 may contribute to Fe-S cluster assembly on DNA polymerases in *Arabidopsis*.

## Discussion

In *Arabidopsis*, the components of the cytosolic iron-sulfur cluster assembly complex, including NBP35, NAR1, CIA1, and CIA2, are essential for embryo development [8, 13, 43], which is consistent with the involvement of Fe-S proteins in various essential cellular processes. Although MMS19 was recently identified as a component of the CIA complex, it is nonessential, and two individual *mms19* null mutants are viable in *Arabidopsis* [8]. MMS19 is a single protein without a homolog in *Arabidopsis*, which excludes the possibility that a homolog is functionally redundant with MMS19. The null mutants of *MMS19* are also viable in yeast [16], whereas the mouse *MMS19* knockout is embryonically lethal [19]. Although visible defects were reported in the *mms19* mutants of yeast and mammals [19, 20], no obvious phenotypic change was observed in the *mms19* null mutant of *Arabidopsis* [8]. We found that the *mms19* null mutants are defective in TGS, DNA repair, and flowering time control (Figs 2A and 2B, 4A–4F and 5A–5H), indicating that MMS19 plays an important role in these processes.

Given the absence of obvious phenotypic differences between the *mms19* null mutant and the wild type, MMS19 may facilitate Fe-S assembly on a small subset of Fe-S proteins. Fe-S proteins are usually associated with Fe-S cluster assembly components *in vivo* [19, 20]. By affinity purification of MMS19, we identified the catalytic subunits of DNA polymerases α, δ, and ε but no other known Fe-S proteins (Table 1). The results suggest that the DNA polymerases α, δ, and ε are probably the major Fe-S protein targets of MMS19 in *Arabidopsis*.

The knockout mutants of the catalytic subunits of the DNA polymerases α and ε are embryonically lethal in *Arabidopsis* [27–29], suggesting that the two DNA polymerases are necessary for viability. Fe-S cluster assembly is essential for the activity of the DNA polymerases α, δ, ε, and ζ in yeast [33], which is consistent with the essentiality of the Fe-S assembly complex [19, 20]. In *Arabidopsis*, the null mutants of the Fe-S assembly proteins NBP35, NAR1, CIA1, and CIA2 are embryonically lethal [8, 13, 43], but two independent *mms19* null mutants are viable and show no obvious phenotypic defects [8]. We propose that MMS19 is not essential for the Fe-S assembly on DNA polymerases but increases the efficiency of Fe-S cluster assembly. We compared the phenotypes of the *mms19* null mutants with those of two previously characterized weak mutant alleles of DNA polymerases α and ε in *Arabidopsis* [30, 32]. Our results demonstrate that the effect of the *mms19* null mutations on TGS, DNA repair, and flowering time (Figs 2A and 2B, 4A–4F and 5A–5H) is comparable to that of the weak mutations in DNA polymerases α and ε. Therefore, our study reveals a functional connection between MMS19 and DNA polymerases in *Arabidopsis*. The results support the notion that MMS19 increases the efficiency of Fe-S assembly on DNA polymerases.

The yeast MMS19 was originally identified as a component of the transcriptional regulator TFIIH complex [14] and was subsequently also found to directly associate with the chromatin-related proteins DOS2, RIK1, and CDC20 [17]. In yeast, MMS19 is involved in various nuclear processes including transcriptional regulation, telomere length maintenance, DNA replication and repair, and heterochromatin silencing [14–17]. However, the functional mechanism of MMS19 in these nuclear processes has been unclear. MMS19 was recently reported to act as a component of the cytosol Fe-S cluster assembly complex in yeast and mammals [19, 20]. Our results demonstrate that the *Arabidopsis* MMS19 contributes to various nuclear processes through its effects on the assembly of Fe-S clusters on DNA polymerases in the cytoplasm. Assembly of Fe-S clusters on the catalytic subunits of DNA polymerases is a prerequisite for the formation of active DNA polymerases in mammals [33]. Our study suggests that, during Fe-S cluster assembly on DNA polymerases, MMS19 may increase the efficiency of the Fe-S cluster assembly, thereby affecting nuclear processes including TGS, DNA repair, and flowering time in *Arabidopsis*.

In *Arabidopsis*, Fe-S cluster assembly was recently found to participate in genome integrity, embryonic development, and epigenetic regulation [8]. The DNA glycosylase ROS1, a nuclear Fe-S protein, excises 5-methycytosine via the base excision repair pathway and then contributes to DNA demethylation [44]. The demethylation activity of ROS1 was impaired when the Fe-S cluster assembly component AE7/CIA2 was disrupted, suggest that Fe-S cluster assembly facilitates the function of ROS1 in DNA demethylation [8]. DRE2 is a conserved protein required for Fe-S cluster assembly at an early stage of the cytosol Fe-S cluster assembly [5, 6]. In *Arabidopsis*, AtDRE2 was found to act in DNA demethylation and maternal gene expression mediated by the ROS1 homolog DME [45]. These reports indicate that the Fe-S cluster assembly machinery plays an important role in DNA demethylation, thereby facilitating transcriptional activation. Although MMS19 was found to act as a component of the Fe-S cluster assembly complex, we found that MMS19 is responsible for transcriptional gene silencing. The involvement of MMS19 in transcriptional gene silencing is related to the function of MMS19 in the Fe-S cluster assembly on DNA polymerases. However, we cannot exclude the possibility that MMS19 also takes effect on ROS1 and/or DME to mediate DNA demethylation and transcriptional activation. Further study is required to understand how MMS19 cooperates with other components of the Fe-S cluster assembly complex to act on different target proteins and thereby affects diverse biological processes.

## Experimental Procedures

### Plant materials and growth conditions

The T-DNA lines Salk_121963 and SALK_147068, which are *mms19-1* and *mms19-2*, respectively, were obtained from the *Arabidopsis* Stock Center. The native promoter-driven full-length genomic *MMS19*, *CIA1*, *CIA2*, and *NAR1* were cloned into the modified *pCAMBIA1305* or *pRI909* vectors and were tagged with *3xMyc* or *3xFlag* at their C-terminal ends. For gene transformation, the plant expression constructs were transformed into the agrobacterium strain GV3101 and then introduced into *Arabidopsis* by the conventional flower-dipping method. The primers used for construction are listed in S5 Table. For plant growth, the seeds were thoroughly washed with sterilized water and sown on MS medium plates. After they were kept for 3 days at 4°C, the plates were transferred to a growth chamber with 16-h-light and 8-h-dark at 22°C. 10-day-old seedlings were transferred to soil and were grown with 16-h-light and 8-h-dark at 22°C to obtain adult plants. For plant crossing, when the plant entered the full-blossom stage, all the six stamens were carefully removed by forceps, and the flowering parent stamen was brought to pollinate the emasculated pistil extensively.

### DNA damage treatment

For methyl methanesulfonate (MMS, Sigma, 129925-5G) treatment, 5-day-old seedlings were transferred to upright MS medium plates containing a range of MMS concentrations and were photographed after the seedlings were grown for 10–14 days. For UV-B treatment, 5-day-old seedlings were moved to new MS medium plates and were treated with UV-B light (UV 256 nm, CL-508.G) for 0, 3 and 5 min. After treatment, the seedlings were grown on the MS medium plates for additional 5–7 days and then photographed.

### Quantitative RT-PCR

Total RNA was isolated with Trizol reagent (Invitrogen) from *Arabidopsis* seedlings and was subjected to quantitative RT-PCR. Quantitative RT-PCR was performed according to the operating protocol (TaKaRa). *ACT7* was used as an internal control. The primers used for quantitative RT-PCR are listed in the S5 Table. The significant difference was determined by Student's t-test.

### RNA-seq analyses

Total RNA was extracted from 14-day-old seedlings of Col-0, *mms19-1*, and *abo4*. The total RNA samples were used to produce RNA libraries for deep sequencing (HiSeq 2000, Illumina). The *Arabidopsis* genome sequences and annotated gene models were downloaded from TAIR10 (www.arabidopsis.org). 45-bp sequences called by the Illumina pipeline were mapped to the TAIR10 Arabidopsis genome. Tophat v2.0.12 was used to align the raw reads to the genome sequences allowing up to two mismatches. Cufflinks (v2.0.1 http://cufflinks.cbcb.umd.edu/) was used to assemble transcripts and calculate transcript abundances. Differences in RNA transcript levels were identified with Cuffdiff. Genes with a significant (p<0.01) and >2-fold change in expression were selected for plotting heat maps using R. The RNA-seq data generated in this study have been deposited in the Gene Expression Omnibus (GEO) database, www.ncbi.nlm.nih.gov/geo (accession no. GSE67669).

### Affinity purification and mass spectrometric analysis

Samples of flowers or seedlings (6–10 g/sample) from transgenic plants were ground in liquid nitrogen and suspended in lysis buffer (50 mM Tris-HCl [pH 7.6], 150 mM NaCl, 5 mM MgCl_2_, 10% glycerol, 0.1% NP-40, 0.5 mM DTT, 1 mM PMSF, and 1 protease inhibitor cocktail tablet/50 ml [Roche]). The suspension was mixed by slowly rotation on a vertical vortex mixer for 15 min at 4°C. The suspension was then centrifuged, and the supernatant was incubated with anti-Flag or anti-Myc antibody-conjugated beads (Sigma, A2220, A7470) at 4°C; after 2.5–3.0 h, the preparation was centrifuged to precipitate the protein complex. The precipitate was washed once for 6 min with 10 ml of lysis buffer and then five times for 5 min per time with 1 ml of lysis buffer at 4°C. Finally, the protein complex was eluted with 3xFlag peptides (Sigma, F 4799) or 0.1 M ammonium hydroxide at pH 11.3 (for Myc antibody-conjugated beads). The elution samples were run on a 10% SDS-PAGE gel and were silver stained with the ProteoSilver Silver Stain Kit (Sigma PROT-SIL1). The whole proteins were purified from the gel and then subjected to mass spectrometric analysis as previously described [46].

### Co-IP and gel filtration

For co-IP, total protein extracts were isolated from about 2–3 g of T2 transgenic plants and incubated with antibody-conjugated beads as described for affinity purification. The beads were washed 5 or 6 times with lysis buffer and boiled in SDS-PAGE sample buffer. The boiled samples were run on SDS-PAGE gels followed by Western blotting assay. For gel filtration, 0.8-g of flowers were ground in liquid nitrogen and suspended in 3 ml of lysis buffer (without NP-40). The samples were centrifuged at 12000 rpm at 4°C. The supernatant was passed through a 0.22-micron filter to remove cell debris and then loaded onto a Superose 6 10/300 GL column (GE Healthcare, 17-5172-01). The elution was collected in a series of fractions (500 μl/fraction). The eluted fractions were run on SDS-PAGE gels for Western blotting assay. A standard prestained protein ladder (Thermo Scientific, 26616) was used for calibration.

### Nuclear and cytoplasmic fractionations

*Arabidopsis* materials (1-g samples) were ground and suspended in protein extraction buffer (20 mM Tris-HCl [pH 7.5], 25% glycerol, 20 mM KCl, 2 mM EDTA, 2.5 mM MgCl2, 250 mM sucrose, 5 mM DTT, 1 protease inhibitor cocktail tablet per 50 ml [Roche]). The suspension was filtered through double layers of Miracloth and centrifuged at 1500 *g* for 10 min at 4°C. The supernatant was centrifuged at 14000 rpm for 15 min to acquire the cytoplasmic fraction in the supernatant and the nuclear fraction in the precipitate. The precipitate containing the nuclear fraction was washed several times with pre-cooled nuclear resuspension buffer NRBT (20 mM Tris-HCl, pH 7.5, 25% glycerol, 2.5 mM MgCl_2_, and 0.2% Triton X-100, 5 mM DTT) until the precipitate changed from dark green to white. A 500-μl volume of pre–cooled NRB2 buffer (20 mM Tris-HCl, pH 7.5, 0.25 M sucrose, 10 mM MgCl_2_, 0.5% Triton X-100, 5 mM DTT, 1 protease inhibitor cocktail tablet per 50 ml [Roche]) was added to the nuclear pellet, which was gently suspended. The suspension was overlaid onto 500 μl of pre-cooled NRB3 buffer (20 mM Tris-HCl [pH 7.5], 1.7 M sucrose, 10 mM MgCl_2_, 0.5% Triton X-100, 5 mM DTT, 1 protease inhibitor cocktail tablet per 50 ml [Roche]) and centrifuged at 16,000 *g* for 50 min at 4°C to collect the pellet. Finally, the pellet was resuspended in 250 μl of protein extraction buffer, and then 250 μl of 2xSDS sample buffer was added for denaturation by boiling; the preparation was then subjected to SDS–PAGE and Western blotting. For quality control, UGPase (UDP-glucose pyrophosphorylase, Agrisera, AS05086) and the histone H3 (Abcam, ab1791) were used as a cytoplasmic and nuclear markers, respectively.

### Enzyme activity analysis

The in-gel activity assays for AO isoforms were performed as reported previously [42].

### Fe content determination and ^55^Fe incorporation assay

For determination of Fe content, 4 g of 14-day-old transgenic seedlings harboring the homozygous *ICU2-Myc* transgene in the wild-type Col-0 and *mms19* mutant backgrounds was ground into a fine powder and suspended in lysis buffer. After the preparation was centrifuged at 4°C and the supernatant was passed through a 0.22-micron filter, the supernatant was incubated with Myc antibody-conjugated beads (Sigma, A7470) for 2.5 h for binding. The beads were thoroughly washed several times, and the Myc-tag coupled protein complex was eluted three to four times with 100 μl of 0.1 M ammonium hydroxide at pH 11.3. The elutions of each sample were combined for Fe content determination. Briefly, the samples were incubated with HNO_3_ (Thermofisher, Trace Metal grade) for digestion and then diluted to a certain volume and mixed vigorously for homogeneity. The samples were then subjected to ICP mass spectrometry (Themo ICP-MS Ⅻ) for Fe content determination by the standard curve method.

^55^Fe incorporation was determined according to a previous method with some modifications [47]. The T3 homozygous *ICU2-Myc* transgenic plants in the wild-type Col-0 background were crossed to the *mms19* mutant to obtain the homozygous *ICU2-Myc* transgenic plants in the *mms19* mutant background from the F2 segregating generation. The homozygous *ICU2-Myc* transgenic plants in the wild-type and *mms19* mutant backgrounds were grown for 1 week on MS medium plates. A 0.5-g quantity of 1-week-old seedlings was transferred to 250-ml conical flasks containing 100 ml of liquid MS medium with one-fourth FeSO_4_^.^7H_2_O; the preparation was gently shaken on a horizontal shaker for 1 day at 22°C. Thereafter, the seedlings were incubated in newly prepared liquid MS medium without Fe ion for 12 h. A 160-μCi quantity of ^55^FeCl_3_ (Perkin Elmer) in 2 ml of 0.1 M sodium ascorbate was then added to the liquid medium, which was incubated for an additional 2 days. The liquid was discarded, and the seedlings were thoroughly washed three times in citrate buffer (50 mM citrate, 1 mM EDTA, pH 7.0) and HEPES buffer (20 mM HEPES, pH 7.4). The seedlings were then collected and stored at -80°C. The target Fe-S protein ICU2-Myc was immunoprecipitated with Myc antibody-conjugated beads (Sigma, A7470). The radioactivity of ^55^Fe was measured by liquid scintillation counting (scintillation liquid: Ultima Gold, Perkin Elmer, 6013321).

## Supporting Information

S1 Fig Characterization of the *mms19* mutants. (A) Diagram of the gene structure of *MMS19*. Black and white boxes indicate exons and untranslated regions, respectively, and horizontal lines indicate introns. T-DNA insertion positions are marked by open triangles. (B) The transcript level of *MMS19* was determined by quantitative RT-PCR. *ACT7* was used as an internal control. Error bars indicate SD.
(PDF)

S2 Fig The transcript levels of transposable elements and DNA repair-related genes in the wild type and the *mms19* mutants. (A) The transposable elements were markedly up-regulated in the *mms19* mutant relative to the wild type. (B) The DNA repair-related genes were up-regulated in the *mms19* mutant relative to the wild type. *ACT7* was an internal control. Three independent experiments were done, and the results from one representative experiment are indicated. Error bars show the SD.
(PDF)

S3 Fig Validation of RNA-seq results by quantitative RT-PCR. Randomly selected co-upregulated genes (A), co-downregulated genes (B), and co-upregulated TEs (C) in *mms19* and *abo4* mutants were used for validation. *ACT7* was used as an internal control. Error bars represent the SD of three technical replicates.
(PDF)

S4 Fig The effect of *mms19* on the expression of DNA repair-related genes in response to MMS treatment. The seedlings were grown on MS medium plates for ~10 days and then treated with 100 ppm MMS for 0, 12, 24, 48, and 72 h. The transcript levels of *XRI* (A), *BCRA1* (B), *RAD51* (C), and *GR1* (D) were determined by quantitative RT-PCR. Error bars indicate the SD.
(PDF)

S1 Table Differentially expressed genes in *mms19* identified by RNA-seq. (XLSX)

S2 Table Differentially expressed genes in *abo4* identified by RNA-seq. (XLSX)

S3 Table Differentially expressed TEs in *mms19* identified by RNA-seq. (XLSX)

S4 Table Differentially expressed TEs in *abo4* identified by RNA-seq. (XLSX)

S5 Table List of primers used in this study. (XLSX)
