# Supplementary figures and images for: The Cytosolic Iron-Sulfur Cluster Assembly Protein MMS19 Regulates Transcriptional Gene Silencing, DNA Repair, and Flowering Time in Arabidopsis
Source: PLoS One. 2015 Jun 8;10(6):e0129137. doi: 10.1371/journal.pone.0129137 (PMC4459967; doi:10.1371/journal.pone.0129137)

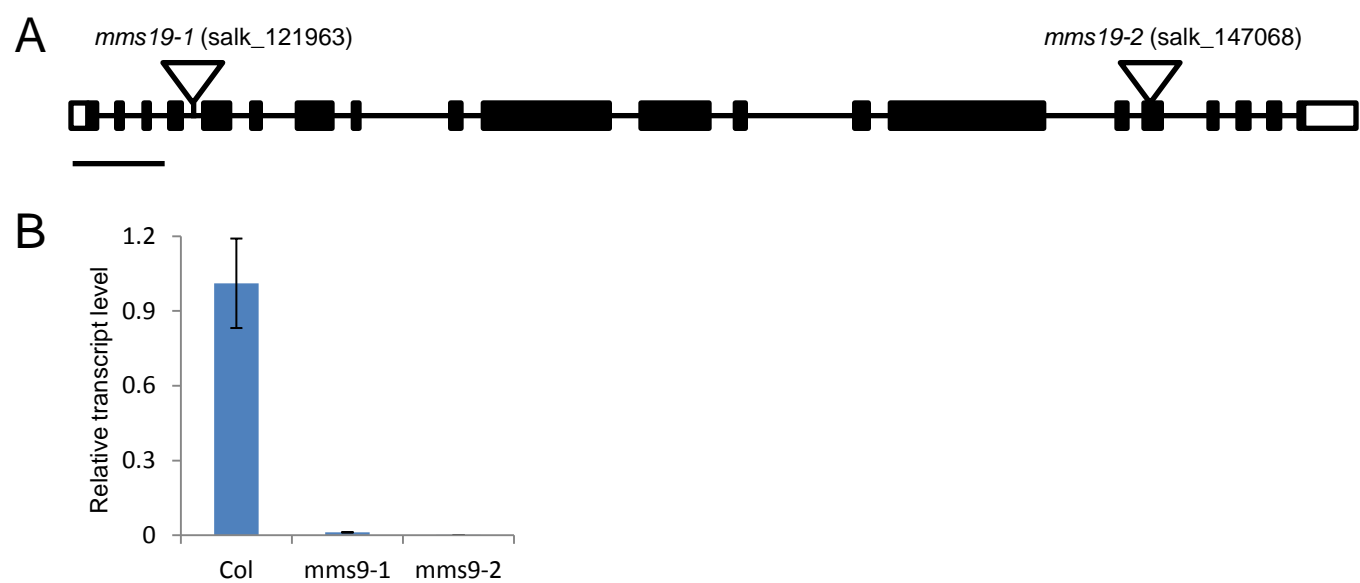

**S1 Fig. Characterization of the *mms19* mutants.**

Supplement: S1 Fig — (A) Diagram of the gene structure of MMS19. Black and white boxes indicate exons and untranslated regions, respectively, and horizontal lines indicate introns. T-DNA insertion positions are marked by open triangles. (B) The transcript level of MMS19 was determined by quantitative RT-PCR. ACT7 was used as an internal control. Error bars indicate SD. (PDF) [file pone.0129137.s001.pdf]

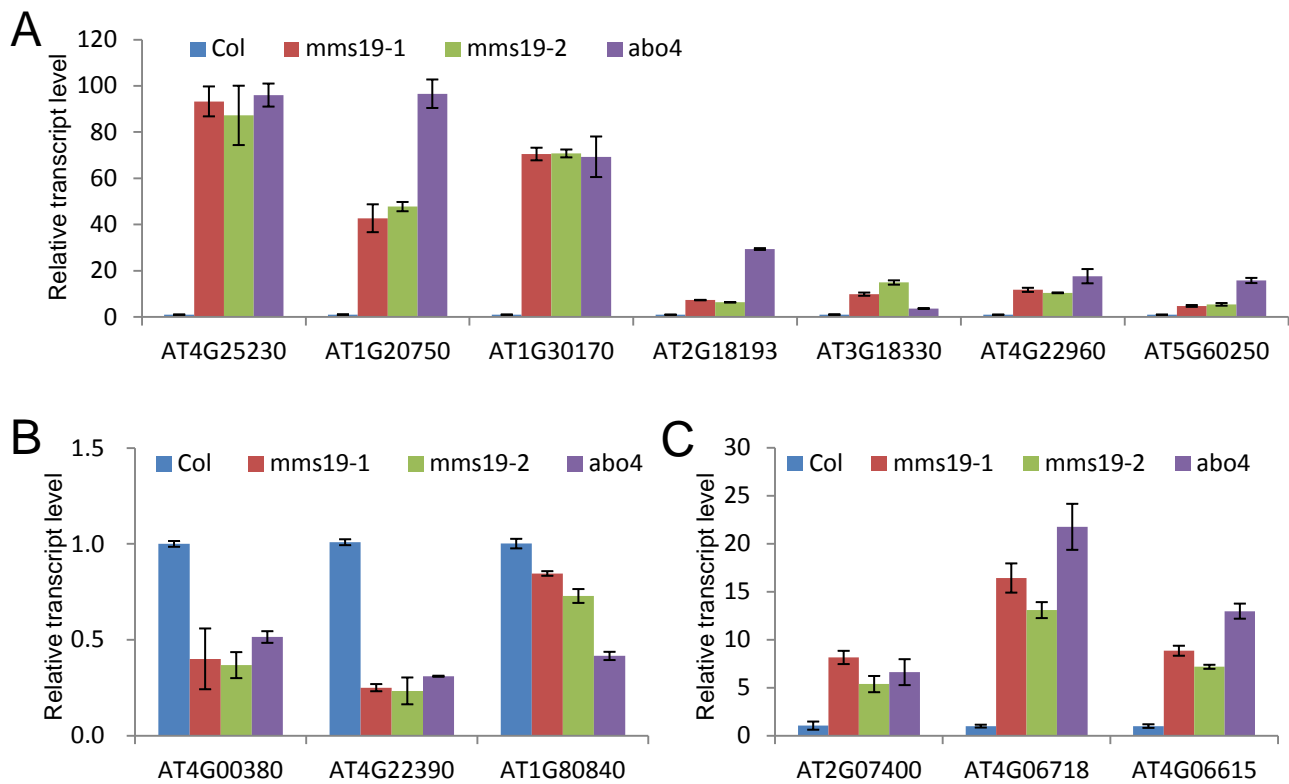

**S3 Fig. Validation of RNA-seq results by quantitative RT-PCR.**

Supplement: S3 Fig — Randomly selected co-upregulated genes (A), co-downregulated genes (B), and co-upregulated TEs (C) in mms19 and abo4 mutants were used for validation. ACT7 was used as an internal control. Error bars represent the SD of three technical replicates. (PDF) [file pone.0129137.s003.pdf]
