# Supplementary material for: The Cytosolic Iron-Sulfur Cluster Assembly Protein MMS19 Regulates Transcriptional Gene Silencing, DNA Repair, and Flowering Time in Arabidopsis
Source: PLoS One. 2015 Jun 8;10(6):e0129137. doi: 10.1371/journal.pone.0129137 (PMC4459967; doi:10.1371/journal.pone.0129137)
Supplement: S2 Fig — (A) The transposable elements were markedly up-regulated in the mms19 mutant relative to the wild type. (B) The DNA repair-related genes were up-regulated in the mms19 mutant relative to the wild type. ACT7 was an internal control. Three independent experiments were done, and the results from one representative experiment are indicated. Error bars show the SD. (PDF) [file pone.0129137.s002.pdf]

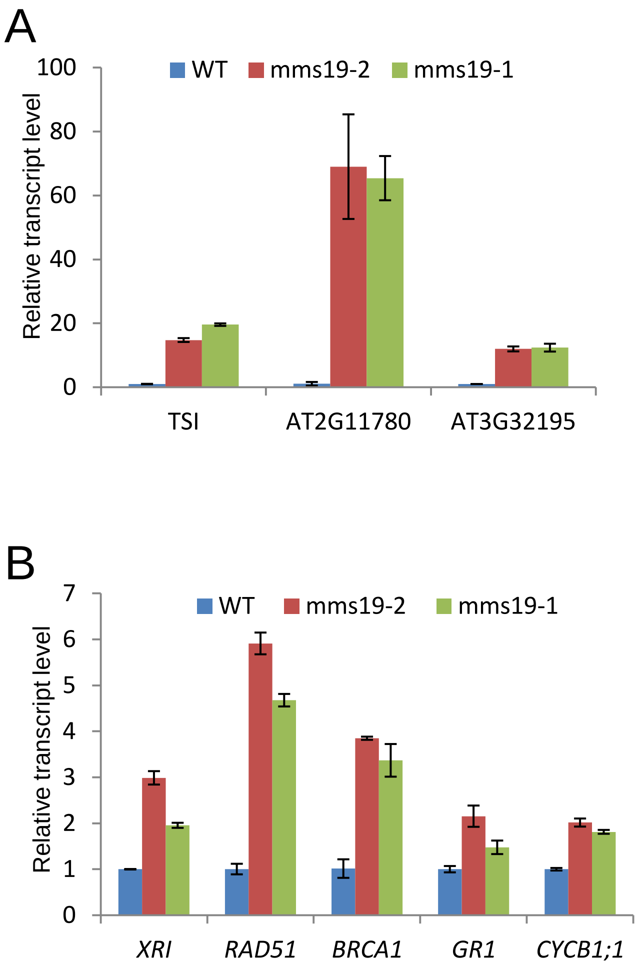

S2 Fig. The transcript levels of transposable elements and DNA repair-related genes in the wild type and the *mms19* mutants.
