# Supplementary material for: The Cytosolic Iron-Sulfur Cluster Assembly Protein MMS19 Regulates Transcriptional Gene Silencing, DNA Repair, and Flowering Time in Arabidopsis
Source: PLoS One. 2015 Jun 8;10(6):e0129137. doi: 10.1371/journal.pone.0129137 (PMC4459967; doi:10.1371/journal.pone.0129137)
Supplement: S4 Fig — The seedlings were grown on MS medium plates for ~10 days and then treated with 100 ppm MMS for 0, 12, 24, 48, and 72 h. The transcript levels of XRI (A), BCRA1 (B), RAD51 (C), and GR1 (D) were determined by quantitative RT-PCR. Error bars indicate the SD. (PDF) [file pone.0129137.s004.pdf]

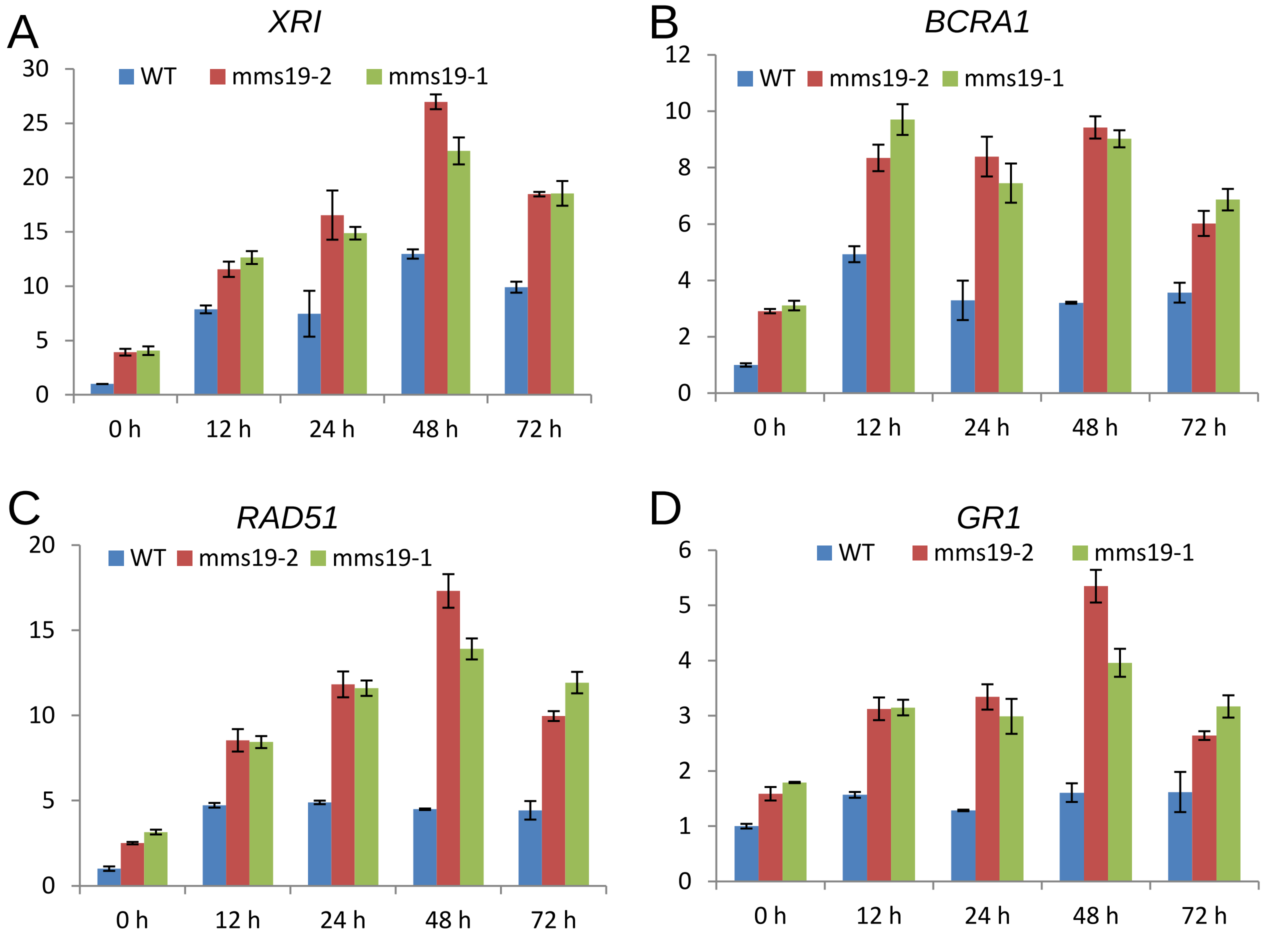

S4 Fig. The effect of *mms19* on the expression of DNA repair-related genes in response to MMS treatment.
